# Clinical and Preclinical Targeting of Oncogenic Pathways in PDAC: Targeted Therapeutic Approaches for the Deadliest Cancer

**DOI:** 10.3390/ijms25052860

**Published:** 2024-03-01

**Authors:** Diego J. Jiménez, Aadil Javed, Teresa Rubio-Tomás, Ndioba Seye-Loum, Carles Barceló

**Affiliations:** 1 Translational Pancreatic Cancer Oncogenesis Group, Health Research Institute of the Balearic Islands (IdISBa), Hospital Universitari Son Espases, 07120 Palma de Mallorca, Spain; 2 Department of Molecular, Cellular, and Developmental Biology, University of Michigan, Ann Arbor, MI 48109, USA; 3 School of Medicine, University of Crete, 70013 Herakleion, Crete, Greece

**Keywords:** PDAC, molecular alterations, oncogenic pathways, targeted therapy, preclinical, clinical, combinational therapy, clinical trials

## Abstract

Pancreatic ductal adenocarcinoma (PDAC) is one of the leading causes of cancer-related death worldwide. It is commonly diagnosed in advanced stages and therapeutic interventions are typically constrained to systemic chemotherapy, which yields only modest clinical outcomes. In this review, we examine recent developments in targeted therapy tailored to address distinct molecular pathway alteration required for PDAC. Our review delineates the principal signaling pathways and molecular mechanisms implicated in the initiation and progression of PDAC. Subsequently, we provide an overview of prevailing guidelines, ongoing investigations, and prospective research trajectories related to targeted therapeutic interventions, drawing insights from randomized clinical trials and other pertinent studies. This review focus on a comprehensive examination of preclinical and clinical data substantiating the efficacy of these therapeutic modalities, emphasizing the potential of combinatorial regimens and novel therapies to enhance the quality of life for individuals afflicted with PDAC. Lastly, the review delves into the contemporary application and ongoing research endeavors concerning targeted therapy for PDAC. This synthesis serves to bridge the molecular elucidation of PDAC with its clinical implications, the evolution of innovative therapeutic strategies, and the changing landscape of treatment approaches.

## 1. Introduction

### 1.1. Background and Significance of Pancreatic Cancer

Pancreatic ductal adenocarcinoma (PDAC) exhibits the lowest 5-year survival rate of all cancers both in the United States and in Europe [1,2,3]. PDAC constitutes the third leading cause of cancer death in men and women in the United states and the seventh worldwide [4]. The current treatment of PDAC is mainly based on surgery and chemotherapy [5,6]. In recent years, surgical techniques have improved, performing venous reconstruction techniques and/or arterial dissection in some cases with vascular involvement [7,8]. In contrast, chemotherapy regimens exhibited a modest improvement with arbitrary physician selection of chemotherapeutic doublets or even triplets combination as the treatment standard [6,9]. Despite these improvements, the overall survival (OS) at 5 years is still <10%, due to the high risk of chemoresistance-driven recurrence [6,9].

This raises the need to translate our understanding of PDAC biology to the clinic to improve survival and quality of life. Here, we review our current knowledge of the molecular features and mutational landscapes of PDAC to develop targeted-based therapies and improve outcomes for patients with PDAC.

### 1.2. Rationale for Targeted Therapies in Pancreatic Cancer

To date, PDAC treatment after resection is based on the usage of chemotherapy which consists of the usage of cytotoxic drugs such as DNA damage agents (oxaliplatin, irinotecan), antimetabolites (gemcitabine, fluorouracil, capecitabine) and cytotoxic balancing agents (leucovorin). FOLFIRINOX (fluorouracil, oxaliplatin, irinotecan, leucovorin) and gemcitabine or its combinations with capecitabine are used for indelicate and delicate-health patients, respectively. In terms of treatment, PDAC can be classified as resectable PDAC, borderline resectable PDAC (BRPC), or locally advanced PDAC (LAPC) based on the degree of tumor contact and involvement with major vessels (superior mesenteric, hepatic artery, or celiac vasculature). According to that, to date, the therapies are based on surgery or not depending on vessel involvement plus cytotoxic drugs. However, the latter are not always effective and do not avoid relapses [10]. Since not all PDAC are resectable, what is being used for these cases are perioperative therapy, i.e., using the agents listed above or the combination of gemcitabine with the mitotic inhibitor albumin-bound paclitaxel (nab-paclitaxel) prior to surgery. The same treatment is used in metastatic PDAC [10]. Unfortunately, chemotherapy is highly unspecific, not always curative and does not avoid relapses: here comes the importance of immunotherapy and targeted therapies which are under investigation. 

Many studies of resected PDAC tumors revealed that KRAS, TP53, CDKN2A and SMAD4 displayed the highest mutation frequencies, with >90% of individuals having oncogenic KRAS mutations. Other genes mutated at a frequency of less than 10%, which included chromatin modification genes (such as ARID1A, KMT2D and KMT2C), DNA repair genes (for example, BRCA1, BRCA2 and PALB2) and additional oncogenes (BRAF, MYC, FGFR1 and others) [11].

Despite the complex mutational landscape, the fact that PDAC tumors become addicted to some oncogenes—i.e., cancer cells exhibit exquisite dependencies on one or several oncogenic drivers to sustain tumor growth and progression [3,11,12,13]—opens the door to new oncogenic addiction-directed therapies to overcome tumor chemoresistance. In fact, most of the patients with available clinical outcomes had an actionable mutation, defined as a genetic aberration for which a specific targeted therapy exists [14]. However, only 7% of these individuals had received a matched, precision-based therapy [15]. Therefore, identifying key oncogenic drivers and dependencies may yield novel approaches for clinical management of PDAC resistance.

Targeted therapies in cancer aim to treat cancer by directly acting on the molecules implicated in the cancer development and maintenance. In this review we will study both therapies that were designed to inhibit a pathway and those that have been taken a step further and allow individuals that have a specific alteration or pre-determined levels of the target to receive the therapy. This motivate the research for new biomarkers that would be used to profile and treat PDAC patients for the sake of precision medicine.

## 2. Molecular Basis of Pancreatic Cancer 

### 2.1. Genetic Mutations and Alterations in Pancreatic Cancer

A common denominator in PDAC is the prevalence of the following four mutations: on the tumor suppressor genes CDKN2A (cyclin-dependent kinase inhibitor 2A, p16 (CDKN2A)), TP53 (tumor protein 53 (TP53)) and SMAD mothers against DPP homolog 4 (SMAD4); and on the oncogene KRAS (Kirsten rat sarcoma viral oncogene homolog (KRAS)) [16]. Additionally, other low frequency occurring mutations in PDAC patients have been discovered in a set of genes that put together happen to be involved in tumorigenesis and tumor maintenance [17,18]. The affected genes and the function alterations are summarized in the following Table 1.

**Figure 1:**
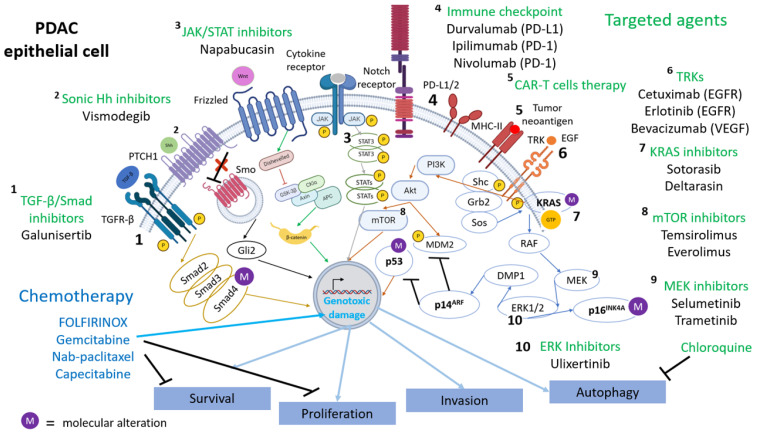
Molecular targets and targeted therapeutic approaches. The main clinically-relevant chemotherapeutic agents and targeted agents are displayed against the main molecular alterations in PDAC, which are KRASG12V, TP53, CDKN2A (p14ARF and p16INK4A) and SMAD4 or their signaling pathways.

Furthermore, these mutated genes converge on the signaling pathways modulated by the following factors: KRAS, TGF-β, WNT, NOTCH, and Hedgehog [3] (Figure 1). These pathways which constitute the hallmarks of pancreatic cancer have components that, in the context of PDAC, are constitutively activated, leading to tumorigenesis and tumor growth maintenance. The high dependency on the mutations mentioned above for PDAC to develop and maintain itself is considered an example of oncogenic addiction. This opens the door to targeted therapeutic interventions as they are considered as potential drug targets. However, there is evidence that they could also be a hurdle while searching for therapeutic targets because of the drug-resistance susceptibility due to their high rate of mutations [19,20].

Cancer cells also rely on normal cellular functions of certain genes that act in oncogenic pathways but are not themselves well-defined canonical oncogenes. This situation is known as non-oncogenic addiction. The non-oncogenes are characterized by not harnessing mutations or genomic alterations making them potential drug targets. This can fortunately help bypass the problems of targeting oncogenic addiction [21] thereby supporting this kind of interventions.

### 2.2. Signaling Pathways Implicated in Pancreatic Cancer Progression

#### 2.2.1. KRAS Signaling Pathway

Mutations in KRAS are among the most common genetic alterations found in pancreatic cancer affecting nearly 90% of PDAC patients [22]. KRAS is a small GTPase which pathway regulation depends on the activation of the Receptor Tyrosine Kinases (RTK) family [23]. In tumor context, KRAS mutation leads to sustained activation of its pathway rendering a transformed state, i.e., cell proliferation, survival and migration. Therefore, the focus of therapeutic development is directed towards directly targeting KRAS through inhibitors or indirectly by targeting its expression rate, membrane location and interaction with effectors [24].

KRAS, a small GTPase, regulates pathways dependent on RTKs activation [23]. It plays a pivotal role in cell functions and affects PI3K/Akt, RAF/MEK/ERK, and RAL-GEF signaling pathways [24]. While considered an oncoprotein, KRAS alone does not cause PDAC and mutations in tumor suppressor genes (CDK2NA, TP53, and SAMD4) are necessary [24]. However, KRAS mutations are the initial genetic alterations in PDAC development, contributing to pancreatic intraepithelial neoplastic lesions (PanIN). Most mutations (82%) occur in codon G12 (G12D or G12V), thereby affecting the catalytic domain. Therapeutic approaches aim to target KRAS directly through inhibitors or indirectly by regulating its expression, membrane localization, and interaction with effectors [24] (Figure 1).

#### 2.2.2. TGF-β Pathway

The activation of TGF-β precursors occurs through proteolytic cleavage, facilitating their binding to type II serine/threonine kinase receptors, which then permits the interaction with type I serine/threonine kinase receptors [25]. Subsequent phosphorylation of the type I receptors leads to the activation of a heterodimer and initiates the SMAD-dependent pathway. This pathway involves phosphorylation of specific proteins known as receptor-regulated SMADs (R-SMADs), including SMAD1, SMAD2, SMAD3, SMAD5, and SMAD8, which form complexes with SMAD4 [25]. These complexes translocate into the nucleus, where they function as transcription factors. Additionally, TGF-β signaling triggers SMAD-independent pathways, such as PI3K/AKT, MAPK, small GTPases like Rac1, NF-κB, as well as activation of pathways like Wnt/β-catenin, Notch, and Hedgehog at the SMAD level [26]. Under normal conditions, TGF-β regulates various cellular processes like cell division, migration, and proliferation [25] (Figure 1).

In a cancer context, TGF-β exhibits a dual role. In the early stages, it acts as a tumor suppressor by promoting apoptosis and restraining epithelial cell proliferation [27]. However, in advanced stages, it facilitates angiogenesis and cell migration by inducing epithelial-mesenchymal transition (EMT). This transition involves the loss of epithelial markers like E-cadherin, enabling invasive behavior of tumor cells, particularly in organs like the liver and lung [28]. In pancreatic cancer, SMAD4 inactivation occurs in nearly half of cases, impairing its anti-tumoral function and promoting pro-tumoral activities. TGF-β receptors, Smad genes, and the selective silencing of TGF-β apoptotic effects by pancreatic tumor cells contribute to this pro-tumoral behavior [29].

Restoring SMAD4 deletion has been observed to inhibit tumorigenic activity in pancreatic cancer cells. Additionally, the combination of SMAD4 inactivation with KRAS mutation worsens cancer prognosis, as evidenced in studies involving mice [30]. Therapies targeting TGF-β, such as inhibitors, chimeric monoclonal antibodies, ligand traps, antisense oligonucleotides, and vaccines, are still undergoing trial phases. These treatments hold promise for managing TGF-β related pathways in cancer [31] (Figure 1).

#### 2.2.3. Hedgehog

The Hedgehog (Hh) signaling pathway plays a critical role in embryonic development but remains dormant in adults. Yet, in certain instances such as injury or disease, limited activation of this pathway is necessary for exocrine pancreas regeneration [32], in pancreatic cancer, this pathway becomes reactivated. 

The Hh signaling pathway consists of various components [32] including Hh proteins (like Sonic Hh–Shh, Indian Hh, and Desert Hh), Patched proteins (Patched 1 and Patched 2), Smo protein, and Gli transcription factors (Gli1, Gli2, and Gli3). (Figure 1) Under normal circumstances without Hh ligand, Patched inhibits Smo, causing Gli2 and Gli3 to act as transcriptional repressors. Conversely, when the Hh ligand is present, inhibition of Smo by Patched is relieved, allowing Gli2 and Gli3 to function as activators, leading to transcriptional activity. 

In pancreatic cancer, approximately 70% of patients exhibit elevated expression of Shh, observed both in early stages (PanIN) and throughout tumor progression. The presence of the KRASG12D mutation correlates with increased Shh expression, worsening prognosis. Additionally, constitutively active NF-kB signaling contributes to elevated Shh levels. Surprisingly, inactivating the Smo gene and the Hh signaling pathway actually intensifies tumor progression, possibly due to compensatory actions by pancreatic tumor cells [33] (Figure 1).

Activation of Smo via Shh produced by pancreatic tumor cells contributes to the production of extracellular matrix components that foster desmoplasia. Studies in mouse models demonstrate that blocking Shh or inhibiting Smo can help reduce tumor growth. However, current anti-hedgehog therapies mainly involve Smo inhibitors, which face challenges like drug resistance and the inability to effectively penetrate the stromal barrier, thus limiting their success in treating pancreatic cancer [33].

#### 2.2.4. WNT 

On basal conditions, β catenin protein is constitutively degraded by the proteasome in the absence of WNT ligands thanks to glycogen synthase kinase 3-beta (GSK3β) phosphorylation activity [34]. The WNT pathway is initiated by the union between frizzled (FZD) receptor, low-density lipoprotein-receptor related protein 5/6 (LRP5/6) and WNT ligand, allowing the sequestration GSK3β promoting the WNT stabilization of proteins (WNT-STOP) process. Thus, degradation of β catenin is prevented and acts as a transcription factor. Sequestration of β catenin also allows activation of the mammalian target of rapamycin signaling induced by WNT (WNT-mTOR). WNT pathway is responsible of regulation of growth, differentiation, and cell death in epithelial cells [34,35,36].

In PDAC, altered expression and activity of upstream or downstream WNT pathway components promote cancer initiation, progression, dissemination, stemness, and therapeutic resistance. It is noteworthy that 5 to 7% of PDAC harbor mutations in ring finger protein 43 (RNF43) mutations conferring growth addiction to WNT ligands through RNF43 inactivation [34]. In fact, RNF43 is a key inhibitor of FZD receptors and LRP5/6 co-receptors through their ubiquitination prior to their proteasomal degradation [34] (Figure 1).

Anti-WNT-based therapies consist of WNT ligands inhibitors and antibodies towards FZD receptors. However, these have shown to be unsuccessful due to side effects such bone compromising [34,36].

#### 2.2.5. NOTCH 

Notch signaling is triggered by the interaction of transmembrane-associated Notch ligand and transmembrane Notch receptor, each factor being present in different cells. Then Notch is successively proteolytically cleaved by the metalloprotease, tumor necrosis factor-α-converting enzyme (TACE) and γ-secretase complex (presenilin and nicastrin). Cleavage by TACE gives Notch extracellular truncation (NEXT) while the of the γ-secretase complex renders Notch intracellular domain (NICD) (Figure 1).

NICD binds to repressor complex mediated by the CBF1, suppressor of hairless and lag-1 (CSL) in the nucleus. In the absence of NICD, transcription of Notch target genes is maintained in an inactive state through CSL. The role of NICD is to displace co-repressors such as SKIP, SHARP, histone deacetylases from CSL. The CSL-NICD complex recruits a co-activator complex containing mastermind, p300, and other co-activators, leading to the activation of Notch target genes such as Akt, ERK, MMP-9, mTOR, NF-κB), p53, etc. (Figure 1).

In pancreatic cancer, this pathway is overactivated in the cancer stem cells (CSCs). Remarkably, CSCs are known to be responsible of tumoural drug-resistant [37]. Anti-NOTCH-based therapies are mainly γ-secretase inhibitors (GSI) and anti-Notch antibodies [38].

### 2.3. Role of Tumor Microenvironment in Pancreatic Cancer Development 

Preceding the development of PDAC, changes occur in the form of pancreatic intraepithelial neoplasias (PanINs) and intraductal papillary mucinous neoplasms (IPMNs) [3]. These histological alterations contribute to the formation of invasive adenocarcinoma by modifying the stroma, thus establishing a favorable environment for cancer progression [39].

In PDAC patients, nearly 90% of the tumor mass comprises a disorganized structure known as desmoplasia within the stroma, consisting of both cellular and acellular components [40]. Cellular elements such as fibroblasts, pancreatic stellate cells, and various immune cells, including tumor-associated macrophages (TAMs) and myeloid-derived suppressor cells (MDSCs), contribute to an immunosuppressive microenvironment, hindering T lymphocyte infiltration and promoting cancer development [41]. Pancreatic stellate cells, exhibiting similarities to myofibroblasts, produce essential extracellular matrix components and signaling molecules [42]. Meanwhile, fibroblasts, particularly cancer-associated fibroblasts (CAFs), collaborate with tumor cells and contribute to the extracellular matrix. The acellular fraction of the stroma encompasses various matrix components like collagen, fibronectin, proteoglycans, and hyaluronan (HA), with the latter associated with poorer prognosis in PDAC cases [42].

The influence of pancreatic cancer cells drives pancreatic stellate cells within the stroma to generate fibrosis, culminating in the formation of desmoplasia. This desmoplasia acts as a barrier, promoting tumor aggressiveness and resistance to drugs [43]. The process of desmoplasia arises from the reactivation of the Hh signaling pathway and the altered expression of extracellular matrix components by stromal cells [18,39]. Additionally, matrix metalloproteinases (MMPs) such as MMP2 and MMP7, released into the extracellular matrix by stromal cells, are associated with increased invasiveness in vitro and in murine models of pancreatic cancer [44,45].

These multifaceted factors create an environment within the tumor niche that impedes chemotherapy, immune cell infiltration, and the efficacy of monoclonal antibodies. Simultaneously, this environment nurtures ideal conditions for pancreatic tumor cell development. It involves processes like angiogenesis, providing nutrients and oxygen to specific parts of the tumor, while also fostering hypoxic regions and limited vascular supply within the tumor core [18,39].

## 3. Targeted Therapeutic Approaches 

### 3.1. Inhibition of RAF/MEK/ERK

Since the discovery of oncogenic drivers, the emphasis in developing therapeutic strategies has been on creating selective inhibitors. This approach has encountered challenges in the case of KRAS oncogenes, as KRAS oncoproteins were traditionally considered virtually undruggable. Nevertheless, the identification of a minute pocket within KRAS, coupled with the potential to establish a stable covalent bond with a mutant cysteine residue, has facilitated the development of the initial selective inhibitors targeting KRASG12C oncoproteins (Figure 1). Notably, this mutation constitutes 13% of all KRAS mutations and is predominantly observed in lung adenocarcinomas (14%) and colorectal tumors (5%) being rare in PDAC (<1%) [46]. Initial findings from phase I/II clinical trials involving the KRASG12C inhibitors AMG 510 or MRTX849 have revealed noteworthy responses in approximately half of lung cancer patients, contrasting with a lack of significant responses in individuals with colorectal tumors [47]. This kind of targeted therapy goes one step beyond traditional targeted approaches for since only individuals that have this very specific alteration receive the therapy.

Proteins acting downstream of KRAS, such as the RAF/MEK/ERK pathway or the PI3K/PDK1/AKT/mTOR pathway, have garnered growing attention. No discernible effect was evident in the clinical trials conducted to assess the effectiveness of MEK inhibitors, specifically selumetinib and trametinib, when administered as monotherapies in patients with advanced PDAC (selumetinib HR = 1.03, 80% CI 0.68–1.57, *p* value = 0.92; trametinib HR = 0.98, 95% CI 0.67–1.44, *p* value = 0.453) [48]. The ineffectiveness of trametinib and selumetinib appears to be attributed to the activation of receptor tyrosine kinases (RTKs). Consequently, clinical trials are underway to investigate the efficacy of multidrug combinations involving MEK inhibitors. High-throughput screening has identified AZD6244 (selumetinib) as exhibiting the highest relative efficacy in PDAC cell lines. Co-administration of AZD6244 with BKM120, a PI3K inhibitor, induces substantial apoptosis in PDAC-derived organotypic models or murine models, leading to an extended median survival (131.5 vs. 71 days). This suggests that the simultaneous inhibition of MEK and PI3K may hold clinical significance. AKT inhibitors also demonstrate robust synergistic effects with MEK inhibitors in PDAC [48].

Ulixertinib, an ERK inhibitor, exhibits inhibitory effects on solid tumor xenograft models, and its combination with MEK inhibitors appears to more effectively impede tumor growth. In summary, strategies targeting both major downstream pathways of KRAS, namely RAF/MEK/ERK and PI3K/PDK1/AKT, concurrently represent a promising avenue for future exploration in the treatment of KRAS-mutant PDAC. Clinical trials have been conducted to assess the efficacy of this combinatorial approach [48].

### 3.2. Inhibition of Other Main Signaling Pathways

#### 3.2.1. PI3K/AKT/mTOR Pathway Inhibitors

The PI3K pathway plays a key role in cancer development and progression by regulating various processes, including T cell differentiation, chemoresistance, angiogenesis, and cell survival. Therefore, using inhibitors of this pathway presents a dual-targeting approach that can impact cancer cells and cancer-associated phenotypes [49]. Akt controls cell growth, division, and survival by triggering the phosphorylation of various proteins that promote cell survival and proliferation, including transcription factors and proteins involved in the cell cycle [50] (Table 2).

Elevated Akt activity has been observed in approximately 60% of PDAC samples due to hyperphosphorylation events, while overexpression resulting from gene amplification has been detected in 10–20% of PDAC patients [51,52]. Inhibitors of the mTOR kinase, such as Everolimus, are used to slow disease progression and enhance the effectiveness of gemcitabine chemotherapy. Using agents that simultaneously inhibit both mTORC1 and mTORC2 has shown improved efficacy compared to targeting a single complex [53]. Sonolisib (PX-866) is a PI3K inhibitor, which is Wortmanin analog, has been used in clinical settings, showed prolonged stable disease for incurable tumors including Pancreatic neuroendocrine carcinoma [54]. It has been used in combination with other drugs in solid tumors and research is ongoing to determine its efficacy [55]. Alpelisib targeting PI3Ka is a promising inhibitor against PDAC as the activity of PIK3a promotes the metastases of the tumors in preclinical studies [56].

A phase I clinical trial of Alpelisib was concluded with safety dosages for the patients receiving combinatorial treatment of nab-paclitaxel and gemcitabine [57]. A pan-PI3K inhibitor named Buparlisib has been tested in phase I clinical trial for various cancers including PDAC and is recommended for further investigations in PDAC due to promising results [58]. Another study utilized Buparlisib with Akt inhibitor and promoter apoptosis in Pancreatic cancer cell lines. Researchers recommended to take into account the target gene mutations while using PI3K/Akt inhibitor [59]. Another study investigated the side effects of Buparlisib in addition to mFOLFOX in metastatis Pancreatic cancer and concluded the tolerated dose of the inhibitor when used in combination with other chemotherapeutic agents [60]. Another PI3K inhibitor Copanlisib displays antitumor capability against advanced tumors including PDAC with action on p110 isoforms [61]. This inhibitor was considered safe for diabetic patients with advanced cancers. Pictilisib inhibiting PI3K has been recommended for phase II trials after promising results in phase I study that included PDAC patients [62]. Further studies have shown that KRAS mutation context is important in determining the outcome of the use of PI3K inhibitors including Pictilisib. However a similar study is yet to be conducted on Pancreatic cancer cells [63]. When Pictilisib was used in combination with Cobimetinib (a MEK inhibitor), the recommended dose was not found due to minimal effieciency with tolerable dosage and the study was discontinued [64]. 

An allosteric Akt inhibitor Perifosine was used in a phase II study for advanced PDAC using Perifosine, but the progression rate of the disease was not reduced [65]. A phase II clinical study reported adverse effects with no positive outcomes for survival in PDAC patients [66]. However, it has shown promising results in different clinical trials for other cancers and leads to prevention of gemcitabine resistance in Pancreatic cancer cells as shown in preclinical studies [67,68]. Another Akt inhibitor Uprosertib was combined with Trametinib in a clinical study involving PDAC patients but showed reduced tolerance without improvement in clinical parameters [69]. Oleandrin targeting Akt has been studied in preclinical settings in a wide assay of Pancreatic cancer cell lines [70] and with PDAC patients in phase I study, it showed reasonable tolerability [54,71]. The phase II study, it showed modest results in metastatic PDAC [71,72]. Its combination with other drugs was recommended. Afuresertib is another Akt inhibitor which has been studied for pharmacokinestics in solid tumors and Phase II study is still underway. Afuresertib has shown antitumor effects and results of the study will help in understanding the effects of the drug further [73]. Archexin is a 20-mer specific inhibitor of Akt and has been employed in a phase II trial for PDAC patients given a prior gemcitabine treatment [74]. The results of the trial are yet to be published. MK-2206 inhibits three forms of Akt together and has shown good results in Pancreatic cancer cell lines, with or without gemcitabine [75]. In patient-derived xenograft models, in combination with Dinaciclib, it reduced the growth of Pancreatic cancer [76]. In a clinical setting, MK-2206 did not show similar results as shown in pre-clinical data [77]. Tricirbine phosphate monohydrate also known as TCN inhibits Akt isoforms and show synergestic effects with gemcitabine in Pancreatic cancer cell lines [31]. mTOR inhibitor Sirolimus in combination with Sorafenib and Sunitinib that inhibit VEGFR kinases, were used in clinical trials for different cancer including PDAC [78]. In the phase II study the overall survival rate was determined to be 6 months with this mTOR inhibitor [79,80]. Everolimus also targeting mTOR directly showed good results in preclinical studies against several cancers. It has been tested in clinical trials with and its monotherapy did not result in positive outcomes as well [81,82]. Therefore, it was recommended that upstream elements of this pathway could be the better targets. It was used in combination with Capecitabane and showed better results in prolonging overall survival [83]. Everolimus a mTOR inhibitor showed good results in phase I study but phase II results showed no improvement for advanced PDAC patients given Everomilus with Octreotide. However, monotherapy with Everomilus had better outcomes alone [82]. Metformin inhibits mTOR via AMPK and has been investigated against Panreatic cancer in combination with other chemotherapeutic agents in a phase II trial where the antitumor activity of chemotherapy were not enhanced upon addition of metformin [83]. Another phase II study involving metformin and other chemotherapy agent Sirolimus concluded that addition of metrormin increases the overall survival [84]. Ridaforolimus is another mTOR inhibitor that was combined with Bevacizumab for advanced Panreatic adenocarcinoma and showed promising results in phase I study with prolonged disease upon administration, however caution was recommended to monitor the side effects [85]. Vistusertib inhibits mTORC1/C2 and is considered a potent agent against PDAC in preclinical and phase I settings with partial response in clinical administration [86]. Temsirolimus is another mTOR inhibitor which showed promise in xenograft models of Pancreatic cancer, however clinical translation resulted in lesser efficacy. It was further combined with Everolimus, but the outcome did not improve with this combination either in the phase II study [87]. In combination with gemcitabine, Temsirolimus resulted is lesser toxicity but the efficacy was not improved [88].

A dual inhibitor of PI3K/mTOR named Dactolisib showed antitumor activity in Pancreatic cancer cells and xenografts, and in combination with gemcitabine showed even better results [89]. It leads to activation of ERK pathway which is associated with tumorigenic phenotypes, therefore, clinical use will depend on this consideration as well [57]. In combination with Sonidegib, a potent combination can be formed inhibiting Hedgehog pathwat as well, therefore signaling pathways diverging at some point can be targeting with combination of different inhibitors targeting multiple pathways [74]. SF1126 is another dual PI3K/mTOR inhibitor which shows promise in targeting advanced malignanies [90]. However further studies are required to recommend it for clinical phase II analysis. Another dual inhibitor for PI3K/mTOR1/C2 is Voxtalisib, which was combined with Primasertib that inhibits MEK, but showed poor tolerance [91]. In monotherapy of Voxtalisib, stable disease was shown as the outcome with tolerable toxicity against solid tumors including PDAC [92]. Omipalisib (PI3K/Akt/mTOR inhibitor) together with Trametinib has shown good results in Pancreatic cancer cells recently. The safety study concluded that Omipalisib is tolerable and therefore shows promise for phase II study [93,94]. Two dual inhibitors of PI3K/mTOR namely PF4691502 and Gedatolisib were administered to patients in phase I study with PDAC patients showing no significant improvement [95]. Despite the development of numerous inhibitors targeting this vital signaling pathway, only a few have demonstrated efficacy in pancreatic cancer in preclinical studies, and even fewer have been investigated in clinical trials. Unfortunately, many of these inhibitors are not yet suitable for patient use due to their toxicity and interference with other essential signaling pathways. Therefore, innovative approaches are necessary to evaluate the clinical effectiveness of these drugs [74,96].

**Table 2:** Targeted therapies in PI3K/AKT/mTOR pathway.

Drug	Target	References
Everolimus	mTOR	[53]
Sonolisib (PX-866)	PI3K	[54]
Alpelisib	PI3Ka	[55]
Buparlisib	AKT	[58]
Copanlisib	PI3K (p110a)	[61]
Pictilisib	PI3K	[62]
Perifosine	AKT	[65]
Uprosertib	AKT	[69]
Oleandrin	AKT	[70]
Afuresertib	AKT	[73]
Archexin	AKT	[74]
MK-2206	AKT	[75]
TCN	AKT	[31]
Sirolimus	mTOR	[78]
Ridaforolimus	mTOR	[85]
Vistusertib	mTORC1/C2	[86]
Temsirolimus	mTOR	[87]
Dactolisib	PI3K/mTOR	[89]
Voxtalisib	PI3K/mTOR	[90]
Omipalisib	PI3K/mTOR	[93]
PF4691502	PI3K/mTOR	[95]
Gedatolisib	PI3K/mTOR	[95]

#### 3.2.2. Wnt/β-Catenin Pathway Inhibitors

The Wnt/β-catenin pathway plays a crucial role in regulating somatic stem cells in various tissues and organs and has been linked to pancreatic cancer development through its control of cell cycle progression, apoptosis, epithelial-mesenchymal transition, angiogenesis, stemness, and tumor immune microenvironment [97]. LGK974 inhibits porcupine enzyme involved in Wnt ligand secretion, and has been used to study its efficacy in Pancreatic cancer cell lines and xenograft model [98]. Also mentioned as WNT974 was recently shown in a phase I trial as well tolerable and indicate that WNT974 may have an impact on the recruitment of immune cells to tumors and may boost the effectiveness of the checkpoint inhibitors [99]. ETC-19221159 another porcupine inhibitor has been tested in Pancreatic cancer cell lines where it has shown reduction in levels of proliferation-related gene expression and accumulation of differentiation markers [100].

Vantictumab (OMP-18R5) antibody inhibits interaction between frizzled receptors and Wnt ligands and in xenograft model of PDAC in combination with gemcitabine showed positive outcomes [97]. In a phase I study vantictumab together with nab-paclitaxel showed stable disease and partial response for progression free survival [101]. Ipafricept is a genetically engineered protein synthesized to contain Fzd8 linked to the Fc domain of human immunoglobulin, and inhibits Wnt signaling by preventing the interaction between Wnt proteins and membrane-bound Frizzled receptors [102]. It exhibited substantial solo efficacy in impeding tumor growth in pancreatic cancer models. Notably, combining paclitaxel with Ipafricept led to a pronounced enhancement of its anti-tumor potency in pancreatic patient-derived xenograft models [103]. During a phase 1b trial, patients with pancreatic cancer that could be evaluated received a combination of ipafricept, nab-paclitaxel, and gemcitabine, and the outcomes showed that ipafricept is safely tolerated in individuals with PDAC [104]. OTSA101 and DKN-01 are other antibodies in clinical trials that can target Wnt pathway in PDAC [105]. PRI-724 targeting beta-catenin, induces the differentiation of cancer stem cells that are resistant to chemotherapy, leading to a reduction in their metastatic potential [106]. The findings of a phase I study suggested that the combination of PRI-724 with gemcitabine can be safely administered to patients with PDAC [107]. Another inhibitor of catenin pathway ICG-001, led to cell cycle arrest in Pancreatic cancer cell lines and showed enhanced survival in xenograft model [108]. Research focused on modulating Wnt signaling holds significant promise for therapeutic advancements. However, rigorous studies are necessary to elucidate Wnt signaling’s role in pancreatic cancer and to thoroughly evaluate the efficacy and safety of Wnt inhibitors, both as single agents and in combination regimens, through robust preclinical testing [34,97].

#### 3.2.3. Hedgehog Pathway Inhibitors

In PDAC, abnormal activation of the Hedgehog (Hh) signaling pathway has been observed, making it a potential target for treatment [109]. Vismodegib targets G coupled receptor smoothened inhibitor (SMO) which is a critical element of Hh signaling. It was combined with Sirolimus in a recently concluded phase I study, showing that combination was well tolerated [110]. A phase II study concluded that the combination of Vismodegib did not work well chemotherapy agents such as gemcitabine and nab-paclitaxel [111]. Another SMO inhibitor Sonidegib was utilized in combination with gemcitabine and nab-paclitaxel and showed tolerance in PDAC patients in a phase II study [112]. Saridegib targeting Hh pathway via SMO revealed mixed outcomes in two studies for PDAC patients with one showing poor effect on survival [113] and the other showed good tolerance in combination with gemcitabine [114]. Another inhibitor belonging to this category known as Taladegib showed no antitumor activity in clinical setting [115]. Furthermore, an antibody MEDI-5304 targeting Sonic hedgehog ligand was developed and showed good antitumor activity in other cancer models but in PDAC, the results were not sufficient to go for clinical evaluation [116]. Similarly, a small molecule inhibitor for Shh Robotnikinin, showed off target cytotoxicity in preclinical studies [117]. Research has focused on creating new compounds that target components downstream of SMO, disrupting both canonical and non-canonical signaling pathways. While promising preclinical results have been achieved, no clinical trials have been initiated due to the restrictive pharmacological characteristics of most compounds [118]. Repurposing drugs that target non-canonical aspects of the Hh signaling pathway may provide a solution to the high failure rate in the process of candidate drug evaluation, potentially leading to swift clinical implementation [109]. 

### 3.3. Angiogenesis and Vascular-Targeted Therapies

The formation of new blood vessels, a process known as angiogenesis, significantly contributes to the advancement of Pancreatic Adenocarcinoma (PDAC) by fostering tumor expansion, invasion, and spread [119]. Pancreatic cancer cells secrete a range of pro-angiogenic factors, including VEGF, PDGF, and FGF, which collectively promote angiogenesis and fuel the growth and spread of the tumor. The formation of new capillaries from existing blood vessels, known as angiogenesis, is crucial for the growth and spread of many solid tumors, including pancreatic cancer, allowing them to proliferate and metastasize. The balance between pro-angiogenic and anti-angiogenic molecules tips in favor of pro-angiogenic molecules, triggering the activation of angiogenesis [120]. Vascular endothelial growth factor A (VEGF-A) stands out among the known pro-angiogenic molecules as a central regulator of abnormal blood vessel growth and maintenance. VEGF-A stimulates vascular endothelial cells to proliferate, promotes their survival, and strongly induces vascular permeability. Additionally, it plays a role in recruiting bone marrow-derived endothelial precursor cells to the site of new vessel formation [67,121]. VEGF-A exerts its pro-angiogenic effects through interactions with VEGFR-1 and VEGFR-2, as well as their co-receptors NRP-1 and NRP-2. Although VEGFR-1 has a stronger binding affinity for VEGF-A, VEGFR-2’s kinase activity plays a more significant role in promoting angiogenesis. As a result, the VEGF-A/VEGFR-2 signaling pathway is considered the primary mechanism driving vessel formation [122]. VEGF-B and PGF, both members of the VEGF family, bind to VEGFR-1, but their functions differ. VEGF-B has a weak angiogenic effect in most tissues and primarily promotes cell survival. In contrast, PGF elicits stronger angiogenic responses in various tissues. This differing activity is unexpected, given their shared receptor interaction. Further research is needed to understand their distinct roles in tumor angiogenesis and progression [123,124]. VEGF-C and VEGF-D primarily promote lymphatic vessel growth (lymphangiogenesis) by interacting with VEGFR-3, distinguishing them from other VEGF family members. Despite their distinct function, they have received relatively less attention regarding their involvement in tumor vessel formation, compared to other VEGF family members [125]. The encouraging results of anti-VEGF therapy in colorectal cancer and other tumor types inspire optimism for the potential application of this anti-angiogenic approach in the treatment of pancreatic cancer, offering a promising avenue to explore [126,127] The effectiveness of anti-angiogenic therapy for pancreatic cancer is a topic of debate. This approach is based on the idea that tumors require new blood vessel formation to grow, and blocking vessel formation can slow tumor growth and benefit cancer patients. While this strategy has extended the lives of many patients, emerging clinical studies suggest that anti-angiogenic therapy may unintendedly promote more invasive tumors and increase the risk of metastasis to distant organs [128,129].

#### 3.3.1. VEGF and VEGFR Inhibitors

Angiogenesis, the formation of new blood vessels, is a vital process that enables cancer to grow and metastasize to other organs. Researchers have extensively explored angiogenesis to understand its significance as a therapeutic target in cancer treatment. Vascular endothelial growth factor (VEGF) plays a key role in angiogenesis, and studies have consistently shown that it is highly expressed in more than 90% of PDAC patients [130] (Table 3).

Brivanib, a tyrosine kinase inhibitor, selectively targets both fibroblast growth factor (FGF) and vascular endothelial growth factor (VEGF). Its anti-angiogenic properties make it a candidate for evaluation in a phase II study for various indications as sole anti-VEGF therapies become increasingly less effective, including the treatment of pancreatic neuroendocrine tumors [131,132]. Aflibercept, also known as VEGF-Trap, is a genetically engineered protein that combines a decoy receptor with a fusion protein. It is designed to prevent angiogenesis by binding to all forms of VEGF-A, VEGF-B, and placental growth factor, thereby inhibiting VEGF-induced angiogenesis [133]. In a phase III trial, it showed less efficacy as compared to Gemcitabine for improving the overall survival in Pancreatic cancer patients [134]. Bevacizumab, a monoclonal antibody that targets human VEGF, has anti-tumor properties. However, a phase III trial conducted from the Cancer and Leukemia Group B (CALGB 80303) found that combining bevacizumab with gemcitabine failed to improve survival rates in advanced PDAC patients, yielding disappointing results [135]. In a phase II trial, it was evaluated the combination of bevacizumab with docetaxel in patients with metastatic pancreatic adenocarcinoma who had received prior treatment. The study found that bevacizumab provided no benefits in gemcitabine refractory metastatic PDAC patients [136]. Sorafenib, a multi-targeting tyrosine kinase inhibitor with anti-angiogenic properties, showed synergistic inhibition of cell proliferation when used individually or in combination with gemcitabine in PC cells [137]. In mouse models of PDAC, researchers found that combining sorafenib and gemcitabine with endothelial monocyte activating polypeptide II (a multi-faceted anti-angiogenic agent) boosted the effectiveness of treatment [138]. A phase III randomized study with a double-blind design found that adding sorafenib to gemcitabine did not improve progression-free survival in patients with advanced pancreatic cancer [139]. Vatalanib, a potent oral inhibitor of multiple tyrosine kinases, exhibits strong affinity for platelet-derived growth factor and vascular endothelial growth factor receptors. In second-line therapy, vatalanib showed good tolerability and led to a notable improvement in 6-month survival rate among patients with metastatic pancreatic cancer [139].

Axitinib is a highly potent and selective inhibitor of VEGF receptors 1, 2, and 3. A phase 2 trial demonstrated improved overall survival in patients with advanced pancreatic cancer, however the combination of Axitinib with gemcitabine fails to enhance overall survival in advanced pancreatic cancer. This finding contributes to the growing body of evidence suggesting that targeting VEGF signaling is an ineffective approach for treating this disease [140,141]. The combination of Axitinib and gemcitabine was well-tolerated, but it failed to demonstrate a survival benefit over gemcitabine alone in patients with advanced pancreatic cancer, both in Japan and other regions [142]. Regorafenib, a multi-kinase inhibitor with anti-angiogenic properties, is being investigated for its anti-VEGF effects [143,144]. It was administered to metastatic PDAC patients who have progressed after gemcitabine chemotherapy, with progression-free survival (PFS) as the primary endpoint in a phase II clinical trial, where it showed minimal activity as a single agent [144]. In a phase II trial, TL118, which exhibits anti-angiogenic properties, is being evaluated in combination with gemcitabine for the treatment of metastatic PDAC. The trial is assessing the efficacy, safety, and tolerability of TL118, and early results in clinical practice suggest encouraging outcomes in terms of progression-free survival (PFS) [145,146]. Foretinib, a multiple kinase inhibitor, has also shown potential in inhibiting tumor growth in vivo by suppressing vascular endothelial growth factor receptor-2 (VEGFR-2) [147]. In combination with Lapatinib it has shown good potential against Breast cancer as well [40]. Cediranib, a pan-VEGF receptor inhibitor has been evaluated for antitumor activity against PDAC cells and provides a rationale to target VEGFR for reducing PDAC burden [148].

**Table 3:** Targeted therapies in VEGF/VEGFR.

Drug	Target	References
Brivanib	FGF/VEGF	[131]
Aflibercept	VEGF-A, B	[133]
Bevacizumab	VEGF	[135,136]
Sorafenib	tyrosine kinase	[137]
Vatalanib	tyrosine kinases	[139]
Axitinib	VEGR1,2,3	[140]
Regorafenib	tyrosine kinases	[143]
Foretinib	VEGFR-2	[147]
Cediranib	Pan VEGFR	[148]
PF4691502	PI3K/mTOR	[95]
Gedatolisib	PI3K/mTOR	[95]

#### 3.3.2. PDGF Inhibitors

Platelet-derived growth factors (PDGFs) and their respective receptors are key contributors to cancer advancement and the development of resistance to chemotherapy especially in PDAC [149] (Table 4). Sunitinib, a tyrosine kinase inhibitor, has shown improvement in progression-free survival (PFS) in the maintenance setting for patients with metastatic pancreatic cancer [150]. It specifically blocks the phosphorylation of vascular endothelial growth factor receptor 2 (VEGFR2) and platelet-derived growth factor receptor β (PDGFRβ) [151]. Nintedanib, an angiokinase antagonist, simultaneously blocks FGFR1/2/3, VEGFR1/2/3, and PDGFRα/β. Preclinical studies have shown nintedanib’s antitumor activity across various cancers. It may serve as an alternative treatment option for pancreatic cancer, either alone or combined with gemcitabine. A clinical trial is currently evaluating the safety and tolerability of combining nintedanib with standard chemotherapy (gemcitabine and nab-paclitaxel) for metastatic pancreatic cancer [152,153]. Imatinib, an inhibitor of platelet-derived growth factor receptors (PDGFR), blocks the PDGFR signaling pathway. When combined with gemcitabine, imatinib exhibits a synergistic effect in treating PDAC, significantly amplifying the anti-tumor effect of gemcitabine in a xenograft model derived from drug-resistant PDAC patient cells [154]. The anti-proliferative effects of Aurora B and pan-Aurora inhibitors were enhanced with the addition of Imatinib as well in Pancreatic cancer cells [155]. Melatonin enhances the anti-cancer effects of sorafenib by suppressing the PDGFR-β/STAT3 signaling pathway and activating melatonin receptors (MT) to inhibit STAT3. This combination may be a promising therapeutic approach for treating PDAC [105]. Masitinib is a multitargeted oral drug that selectively inhibits various protein tyrosine kinases, including c-Kit, PDGFR-3, Lyn, and FAK, making it a precise and potent treatment option [156]. Although masitinib has not demonstrated improvements in overall survival or progression-free survival compared to standard treatment in patients with pancreatic adenocarcinoma, it may still have a beneficial effect on reducing inflammation in patients experiencing increased pain levels related to the disease [157]. TK1258 also known as Dovitinib which inhibits multiple kinases also impairs the FGF1, FGF2, VEGFA, and PDGFB in Pancreatic cancer cells [158]. This inhibitor works on mediating the vasculature as shown in in vivo data. 

### 3.4. Immune Checkpoint Inhibitors in Pancreatic Cancer

Among the identified immune checkpoint proteins, the mechanisms of CTLA-4 and PD-1/PD-L1 inhibition of T cell function are well characterized and understood [159]. Immune checkpoints serve a crucial function by interacting with antigen-presenting cells (APCs) and other cell types to prevent excessive T cell activation. Naive T cells are activated by a dual signaling process involving the T cell receptor (TCR) recognizing antigens and CD28 binding to B7 molecules (CD80/CD86) on antigen-presenting cells. Meanwhile, CTLA-4 expression is induced on naive T cells upon antigen activation and is persistently expressed on regulatory T cells (Tregs) to regulate immune responses [160]. Unlike CTLA-4, PD-1 is expressed on activated T cells, B cells, and myeloid cells. When PD-1 binds to its ligand PD-L1, it sends inhibitory signals to T cells, promoting peripheral immune tolerance by dampening their activity [161]. CTLA-4, a molecule like CD28, binds to CD80/CD86 with a 20-fold higher affinity, thereby blocking T cell activation through CD28. Additionally, CTLA-4 on regulatory T cells (Tregs) can eliminate CD80/CD86 from antigen-presenting cells, impairing their ability to activate naive T cells [162]. Treg cells suppress dendritic cell function via CTLA-4, which binds to CD80/86 and inhibits T cell activation. Tumors evade the immune system by aberrantly expressing PD-L1. But, administering anti-CTLA-4 and anti-PD-1/PD-L1 monoclonal antibodies can reverse these inhibitory mechanisms, enabling T cells to target the tumor. Just like normal cells, cancer cells are attacked by activated T cells after dendritic cells recognize neoantigens. This knowledge led to the approval of various immune checkpoint inhibitor monoclonal antibodies for different cancers [163].

#### 3.4.1. PD-1/PDL-1 Inhibitors

Pembrolizumab, an anti-PD-1 immune checkpoint inhibitor, is the sole immunotherapy approved by the FDA for treating advanced PDAC in patients whose tumors are either mismatch repair deficient (dMMR) or microsatellite instability high (MSI-H) and with no other alternative options available [164,165] PDAC has been considered resistant to immunotherapy due to several challenges within the tumor microenvironment. To overcome these obstacles, researchers have logically explored combining anti-PD-1/anti-PD-L1 checkpoint inhibitors with other immune and targeted therapies. However, a recent phase II randomized clinical trial yielded negative results when testing dual immune checkpoint blockade in advanced PDAC patients [165]. It was a phase II randomized clinical trial investigated the effectiveness of durvalumab, a PD-L1 antibody, either alone or in combination with tremelimumab, an anti-CTLA-4 antibody, in patients with recurrent or metastatic PDAC. Ongoing studies are exploring the combination of PD-1/PD-L1 inhibition with other treatments in PDAC. A phase I study combined nivolumab (anti-PD-1 antibody) with mogamulizumab, an anti-CC chemokine receptor 4 antibody, in patients with advanced solid tumors, including PDAC. The results of the trials should be considered in the context of phase I study. Additionally, innovative CAR T-cell therapies are being developed in preclinical research to target PDAC [166,167].

#### 3.4.2. CTLA-4 Inhibitors

Two early studies utilizing Ipilimumab targeting CTLA-4 in Pancreatic cancer patients showed mixed response [168,169]. Combining anti-CTLA-4 and PD-1 therapies, which target different stages of the immune response, has shown promise. Specifically, using nivolumab and ipilimumab together resulted in longer progression-free survival and a higher response rate compared to ipilimumab alone. However, this combination also led to increased toxicity [170]. Investigations into the pancreatic tumor microenvironment will pave the way for a new treatment approach that combines immune checkpoint inhibitors and chemotherapy along with cancer vaccines to tackle PDAC. Overcoming current challenges in both preclinical and clinical studies will be crucial to demonstrating the effectiveness of utilizing immune checkpoint inhibitors in treating PDAC [11,171].

## 4. Clinical Trials and Current Treatment Landscape 

### 4.1. Overview of Recent and Ongoing Clinical Trials

In contrast to many other prevalent tumours, beyond multiagent chemotherapy, no novel paradigm-shifting therapies have been developed in the last 40 years [172]. Remarkably, a very small proportion of the ongoing and completed clinical trials for pancreatic cancer (https://clinicaltrials.gov/search?cond=Pancreatic%20Cancer [accessed on 10 December 2024]) assess the beneficial effects of targeted therapies. Importantly, most of these biology-based drugs are repurposed, meaning that they are existing drugs already approved for a different indication, which, in most of the cases, is a type of cancer other than pancreatic cancer [172].

Examples of drug repurposing in pancreatic cancer are a plethora of small molecules inhibiting multiple kinases in an unspecific manner (including Sorafenib or Regorafenib), or small molecules and even monoclonal antibodies specifically decreasing the activity of well-known growth-promoting pathways (such as mTOR and Hedgehog pathways), or, instead, promoting cellular senescence. Additionally, vaccines have arisen as a promising therapeutic approach for pancreatic cancer. Nevertheless, even when targeted therapies are evaluated, in most cases they are administrated to patients in combination or after traditional chemotherapy, with the expected limitations due to toxicity [64].

### 4.2. Targeted Therapies in Combination with Standard of Care

Gemcitabine monotherapy was established as the standard of care in 1997 and, together with FOLFIRINOX (an acronym that stands for the combination of the following drugs: FOLinic acid [also known as Leucovorin], Fluorouracil [5FU], IRINotecan and OXaliplatin), it is still recommended as first-line pharmacological treatment of advanced pancreatic cancer in the latest version of the European Society for Medical Oncology (ESMO) Clinical Practice Guidelines. Second-line and third-line treatment are common chemotherapy (such Cisplatin and Paclitaxel) with a striking absence of targeted therapies. Other relevant guidelines, such as those from the Japan Pancreas Society [64] recommend similar therapeutic approaches.

In addition, as aforementioned, targeted therapies in clinical trials are scarce in comparison with the advances towards precision medicine in other cancer types and, most importantly, most targeted therapies are not tested in clinical trials alone but in combination with standard of care. Nevertheless, some targeted approaches are under study.

Remarkably, more than 10 clinical trials evaluating the administration of Bevacizumab (a Vascular endothelial growth factor [VEGF] inhibitor, e.g., NCT03351296) and another 10 clinical trials using Cetuximab (an Epidermal growth factor receptor [EGFR] inhibitor, e.g., NCT00042939) have been registered in the last 5 years, indicating a growing interest for these directed therapies in pancreatic cancer.

Multi-kinase inhibitors have also been proposed as directed therapies for many cancer types, including pancreatic cancer. Sorafenib is a multi-kinase inhibitor that mainly targets Raf-1 kinase (a member of the RAF/MEK/ERK signaling pathway), VEGFR2, and PDGFR-β, restricting tumour cell proliferation and angiogenesis [135,141]. In the last decade, there have been many efforts to test its utility as a treatment for pancreatic cancer [141]. Indeed, a Phase I study assessing the safety and tolerability of Sorafenib in combination with Gemcitabine and radiotherapy in patients with unresectable pancreatic cancer (NCT00375310) has been completed recently. Furthermore, there is an ongoing Phase II trial combining Sorafenib and Vemurafenib (an inhibitor of the serine-threonine kinase BRAF carrying the activating mutation V600E), that elicits downstream activation of the RAS/RAF/MEK/ERK mitogen-activated protein kinase [MAPK] signalling pathway, in patients with KRAS mutated pancreatic cancer who have progressed on standard chemotherapy (NCT05068752). Interestingly, the combination of Sorafenib and the HDAC inhibitor Vorinostat (targeting epigenetic dysregulation) was well tolerated by patients with pancreatic cancer after neoadjuvant chemotherapy widening the horizons for a combination of standard of care, multikinase inhibitors and drugs targeting the tumour epigenome.

Similarly, the small-molecule multi-kinase inhibitor Regorafenib has been tested as a single agent in patients with metastatic pancreatic cancer that have progressed after treatment with gemcitabine and for which no other standard treatment options exist (NCT02080260, NCT02259725).

Regarding immunotherapy, several clinicals trials evaluating Toripalimab (a recombinant humanized monoclonal antibody against programmed cell death protein 1 (PD-1) that acts as a checkpoint inhibitor [145] in combination with chemotherapy are currently active and/or recruiting participants (such as NCT05934331, NCT05580445 and NCT06111274).

In the same line, the efficacy of activated T-lymphocyte cell therapy was assessed in a small group of patients with Gemcitabine-refractory advanced pancreatic cancer. This Phase II trial included isolation and activation of T-lymphocyte from subject’s blood and intravenous administration for 1 h, with low rate of adverse effects but modest improvements in overall survival and disease progression (NCT00965718). Efficacy and dosage of bispecific antibody-armed activated T cells are also under study (NCT04137536 and NCT03269526).

Immune checkpoint inhibition can also be achieved with Tremelimumab (a human monoclonal antibody that blocks the activity of cytotoxic T-lymphocyte-associated protein 4 [CTLA-4]). Tremelimumab, in combination with radiotherapy and Durvalumab (a human monoclonal antibody against programmed death-ligand 1 [PD-L1]), has been tested in patients with pancreatic cancer that was unresponsive to chemotherapy (NCT02311361).

Promising clinical trials with inhibitors of the mTOR pathway, including Temsirolimus (NCT00075647), Sirolimus (also known as rapamycin, NCT00499486) and Everolimus (NCT02305810) are also on going, mainly for patients with advanced and metastatic pancreatic cancer. In addition, a Hedgehog inhibitor, in combination with chemotherapy (Gemcitabine and nab-paclitaxel]), has been examined in a Phase II trial in patients with metastatic pancreatic adenocarcinoma (NCT01088815).

Since senescent cells are cell-cycle arrested, the induction of cellular senescence in tumoural cells is a widely used approach for the treatment of many cancer-types, despite its detrimental long-term effects [105]. In this line, Palbociclib (a CDK4/6 inhibitor inducing cellular senescence) has been administrated to patients with metastatic PDAC, in combination with nab-paclitaxel in a Phase I study (NCT02501902). Although it seems to be well-tolerated, another study combining Palbociclib and the selective inhibitor of MEK1/MEK2 Trametinib does not show promising results for this combination as an effective later-line treatment for metastatic PDAC [22].

In the forefront of the newest therapeutic strategic are vaccines. The safety and efficacy of neoantigen peptide-based vaccines is being examined in patients with PDAC, among others (NCT03558945). The emergence of mRNA vaccines as therapeutic approaches has led to a recently started clinical trial in combination with blockade of Programmed Death-Ligand 1 (PD-L1) (NCT04161755). Moreover, cancer stem cells have been shown to be immunogenic (i.e., capable of promoting protective antitumor immunity) when inoculated into immunocompetent mice. Based on this rationale, a study has been designed to determine the feasibility of generating cancer stem cells vaccines by loading dendritic cells with patient’s pancreatic cancer stem cells that can activate cytotoxic T cells and, thereby, be used in the clinics (NCT02074046).

### 4.3. Challenges and Limitations in Clinical Implementation

Previous clinical trials in pancreatic cancer led to minimal improvements in patient’s survival [172]. Indeed, clinical management guidelines for pancreatic cancer have not changed for decades and do not include any targeted therapy. Therefore, considering that the average survival of pancreatic cancer in Europe is the lowest of all cancer types and that many patients present unresectable or metastatic disease [145], any progress can be considered an important milestone in the treatment of this deadly cancer (Table 5).

## 5. Emerging Trends and Future Directions 

### 5.1. Novel Targeted Agents in Preclinical Development

Owing to the limited efficacy of existing PDAC treatments, there is a pressing need to explore fresh therapeutic targets specific to this subtype of pancreatic cancer. Numerous aspects contributing to the tumor’s resistance to treatment, including epigenetic modifications, DNA repair mechanisms, the tumor microenvironment (such as the extracellular matrix and immune system), and autophagy, can be leveraged for the identification of novel therapeutic avenues. A subset of PDAC patients, approximately 10–15%, exhibits DNA repair pathway alterations beyond BRCA, representing potential pharmacological targets [41] (Table 6).

While immune system-based therapies have shown efficacy in liquid tumors, physical barriers and an immunosuppressive environment in solid tumors hinder the penetration of monoclonal antibodies or CAR-T therapies into the tumor microenvironment [11]. However, ongoing research presents promising advancements, like vaccination strategies aiming to sensitize and revitalize the immune system against pancreatic tumor cells [3]. 

For instance, a vaccination method involving the expression of granulocyte-macrophage colony-stimulating factor (GM-CSF) in irradiated pancreatic tumor cells recruits dendritic cells to the tumor site, fostering cytotoxic T cell activation [3]. Chloroquine, an autophagy inhibitor, disrupts the tumor cell’s escape mechanism from apoptosis, enhancing the sensitivity of tumor cells to certain therapies like gemcitabine, MEK inhibitors, or nab-paclitaxel [3]

The SWI/SNF complex genes, frequently altered in PDAC patients, play a significant role in tumorigenesis [173]. Deletion of specific complex factors, such as Arid1a or Brg1, can promote tumor development, although their role in cancer is multifaceted. Notably, the complex’s epigenetic modifications drive tumor plasticity, responsible for chemoresistance [3]. Interestingly, this plasticity influences tumor responsiveness to MEK inhibitors when patients are treated with FOLFIRINOX as a neoadjuvant therapy (Table 6). 

Moreover, the action of TGF-beta, especially in PDAC patients with mutated KRAS, drives tumor plasticity. Inhibiting TGF-beta’s effect on pancreatic cells or its signaling pathway emerges as a potentially significant therapeutic approach, considering the high prevalence of KRAS mutations in patients [28]. However, TGF-beta exhibits a dual role in tumor progression, acting as a suppressor in early stages but promoting EMT in advanced PDAC stages. Clinical trials are exploring inhibitors targeting TGF-beta signaling, such as galunisertib, particularly for patients with metastatic or locally advanced PDAC [28]. 

Additionally, studies propose that statin-induced inhibition of the cholesterol signaling pathway could improve PDAC patient survival by regulating TGF-beta expression, suggesting cholesterol as a metabolic trigger influencing EMT and potentially serving as a therapeutic target in PDAC treatment strategies [174].

**Table 6:** Novel targeted therapies in clinical trials.

Drug or Intervention	Target	Phase of Trial	Preclinical References	Clinical References
adjuvant chemotherapy FOLFIRINOX	folate reductase, thymidylate synthetase, topoisomerase I, DNA	NA	NA	[41]
Gemcitabine	topoisomerase I	NA	NA	[41]
Gemcitabine + capecitabine	thymidylate synthetase	NA	NA	[41]
Gemcitabine/nab-paclitaxel + Pembrolizumab	PD-1	NA	NA	[173]
Sotorasib	KRASG12C	I/II	NA	[24]
Irinotecan + 5-FU	topoisomerase I, thymidylate synthetase	NA	NA	[173]
Adoptive T cell	HLA-restricted mutant KRAS neoantigen	NA	NA	[173]
Exosome-delivered synthetic siRNAs	KRASG12D	NA	NA	[173]
Autologous CAR-T cells	B7-H3 antigen	I	Clinical trials	NA
Vismodegib + chemotherapy	SMO	NA	NA	[173]
TAMs (novel agonists) + pembrolizumab	CD11b/CD18 + PD-1	NA	[173]	NA
Gemcitabine + CD40 agonists	CD40	NA	[173]	NA
Gemcitabine + erlotinib	EGFR	NA	NA	[3]
Gemcitabine + TH-302	DNA	NA	NA	[3]
MM-398 + 5-FU + folinic acid		NA	NA	[3]
Deltarasin	KRAS	NA	NA	[3]
Chloroquine or hidroxychloroquine	Lysosomes	NA	[3]	NA
GVAX pancreas	pancreatic tumor cells	NA	[3]	NA
Gemcitabine/or nab-paclitaxel + Ipilimumab	CTLA-4	NA	NA	[3]
Durvalumab + tremelimumab	PD-L1 + CTLA-4	II	NA	[11]
FOLFIRINOX + CCR2 inhibitors	CCR2	NA	[175]	NA
CCR2 inhibitors + Nivolumab + chemotherapy	PD-1	NA	NA	[11]
CD40 agonist + FLT3L	CD40 + Tyrosine kinase 3	NA	NA	[11]
CD40 agonist + FLT3L	CD40 + Tyrosine kinase 3	NA	NA	[11]
LCL-161	ABCB1-ATPase/ABCB1	NA	[175]	NA
DRI-C21045 + anti-PD1 + GnP	CD40	II	NA	[11]
Gemcitabine + everolimus + thermosensitive hydrogels	mTOR	NA	[31,144]	NA
GSH + UNC0638	GSS + EHMT2	NA	[37,75]	NA
Gemcitabine-nab-paclitaxel + ATRA	RARs (RAR-α, RAR-β, RAR-γ)	II	NA	[176]
Losartan + chemotherapy + nivolumab	AT1R	II	NA	[176]
FG-3019+ gemcitabine-nab-paclitaxel or FOLFIRINOX	CTGF (connective tissue growth factor)	NA	[176]	[176]
Gemcitabine + Napabucasin	STAT3	NA	[176]	NA
64Cu-DOTA-ECLli	CCR2	I	NA	Clinical trials
1ºAzacitidine and/or Romidepsin + nab-paclitaxel/Gemcitabine	DNMT/HDAC	I/II	NA	Clinical trials
2ºDurvalumab + low-dose Lenalidomide	PD-L1/TNF-α, IL-1β, IL-6 and GM-SCF	I/II	NA	Clinical trials
nab-Paclitaxel/Gemcitabine + Camrelizumab/Radiotherapy	PD-1	Observational	NA	Clinical trials
Zimberelimab/SBRT + quemliclustat and/or etrumadenant	PD-1/CD73/A2a or A2b	II	NA	Clinical trials

### 5.2. Combination Strategies for Enhanced Efficacy 

The concept of merging drugs emerged as a response to combat acquired chemoresistance in tumors. This resistance stems from new mutations that grant protection against conventional therapies. Monotherapy using current drugs falls short in treating PDAC, prompting the exploration of combined drug approaches and preclinical trials targeting newfound avenues [173].

The primary treatment for metastatic PDAC patients involves administering a combination of 5-fluorouracil (5-FU), leucovorin, irinotecan, and oxaliplatin collectively known as FOLFIRINOX (an acronym that stands for the combination of the following drugs: FOLinic acid [also known as Leucovorin], Fluorouracil [5FU], IRINotecan and OXaliplatin). However, despite its efficacy in extending life expectancy and progression-free survival compared to gemcitabine, its usage has been associated with notable side effects [41]. FOLFIRINOX serves as adjuvant chemotherapy post-PDAC resection for non-metastatic tumors. Researchers have developed platforms to exploit vulnerabilities induced by treatments, exemplified by MEKi sensitivity due to tumor plasticity arising from FOLFIRINOX exposure [177].

Nevertheless, even with these advancements, achieving complete patient recovery remains elusive. In cases where the first-line treatment fails [173] the second-line comprises gemcitabine combined with nab-paclitaxel or a liposomal formulation of irinotecan in combination with 5-FU, designated as neoadjuvant therapy for locally advanced pancreatic cancer (LAPC). 

**Figure 2:**
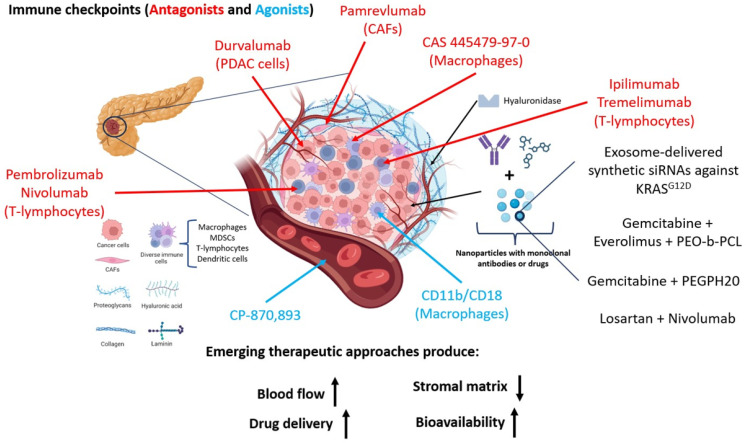
Emerging therapeutic approaches. The schematic illustration portrays the intricate milieu of a tumor microenvironment, delineating the extracellular matrix and its pivotal constituents, namely hyaluronic acid, collagen, laminin, and proteoglycans, alongside various cellular elements such as TAMs (tumor-associated macrophages), dendritic cells, T lymphocytes, MDSCs (myeloid-derived suppressor cells), and CAFs (cancer-associated fibroblasts). Within this context, emphasis is placed on elucidating the principal molecules governing immune cell regulation, including CTLA-4, PD-1, and corresponding therapeutic interventions targeting these molecules. The evolution of novel therapies stems from the imperative to breach the stromal barrier, leading to amalgamated formulations encapsulated to enhance targeted therapeutic efficacy; exemplified by the combination of gemcitabine and PEGPH20 (hyaluronidase) confined within nanocapsules.

Preclinical investigations focus on combining gemcitabine with other drugs like everolimus [178] a rapamycin-derived mTOR inhibitor, or capecitabine, recommended for patients with poor functional status [41]. Additionally, exploring various drug combinations unveils a therapy window yet to be fully explored in clinical settings. For instance, trials incorporating Losartan, an angiotensin II receptor agonist, alongside immunotherapy or pamrevlumab with gemcitabine-nab paclitaxel or FOLFIRINOX in patients with locally advanced PDAC are ongoing (Table 6). Approaches involving CD40 agonists with gemcitabine to activate macrophages in the tumor or reprogramming stellate cells to reduce extracellular matrix and tumor growth, are also being developed [3] (Figure 2).

Combining galunisertib with gemcitabine demonstrates enhanced overall survival compared to galunisertib alone, currently undergoing phase 2 and 3 trials. FDA-approved strategies involve pairing gemcitabine with erlotinib, an EGFR tyrosine kinase inhibitor, which marginally increases survival in patients when compared to gemcitabine monotherapy [28] (Figure 2).

### 5.3. Advancements in Drug Delivery for Targeted Therapies

The heterogeneity within tumors, coupled with factors arising from uncontrolled tumor cell growth, such as limited blood flow leading to hypoxic areas, the dense, disorganized stroma, and factors released by CAFs and stellate cells, collectively create a formidable barrier in the tumor microenvironment that impedes drug access. Addressing these challenges represents a major hurdle in PDAC therapy, particularly in ensuring drugs reach the tumor core, where persistent CSCs promote resistance to therapies and foster adaptations resulting in chemoresistance. Consequently, novel efforts are underway to develop effective chemotherapy delivery systems, targeting blood flow, drug delivery and bioavailability while decreasing stromal matrix notably nanomedicine-based therapies [178].

These therapies share common characteristics: the encapsulating structure should enhance drug half-life and bioavailability within the tumor, possess a small size to penetrate the desmoplastic barrier, promote drug accumulation in the tumor, all while minimizing side effects and maximizing therapeutic efficacy. Innovative therapies involve polymers carrying gemcitabine and napabucasin, a STAT3 inhibitor (Table 6). Gemcitabine, an inhibitor of topoisomerase I, serves as a first-line adjuvant chemotherapy for advanced PDAC [178] while STAT3, a transcription factor associated with CSC renewal, interacts with the CD44 receptor overexpressed in tumor cells. Polymers incorporating iRGD sequences target cancer stem cells and deliver the drug to the tumor site [176] (Figure 2).

Polymeric nanoparticles exhibit superior performance compared to lipid-based nanoparticles due to their labile nature, which can impact molecule stability [178] Amphiphilic diblock copolymers like Poly(ethylene oxide)-block-poly(ε-caprolactone) (PEO-b-PCL) conjugated to oxaliplatin, an antitumoral agent, showcase promise in PDAC treatment [37]). Additionally, therapies involving albumin-loaded paclitaxel (nab-paclitaxel) or thermosensitive hydrogels offer solutions to solubility challenges, enhancing drug delivery efficiency [178]. 

PDAC tumors exhibit numerous epigenetic alterations, including DNA methylation, histone acetylation, and chromatin remodeling, activating oncogenes or inactivating tumor suppressor genes. Furthermore, increased glutathione (GSH) levels in PDAC patients create an ideal environment for tumor progression. Studies suggest that administering histone methyltransferase inhibitors may reduce tumor progression and GSH levels, potentially improving PDAC patient prognosis [37,75]) (Figure 2).

## 6. Conclusions

The last translational approaches have elucidated specific vulnerabilities in PDAC and have enhanced our comprehension of the tumor microenvironment. As evidenced by the recent successful approvals of PARP inhibitors and PD-1 blockade for molecularly defined subclasses of PDAC, precision-oriented and immunotherapy-based preclinical and clinical pipelines are paving the way for novel therapeutic avenues. Particularly promising are the ongoing advancements in KRAS-specific inhibitors for PDAC. As observed in other prevalent malignancies, upon the introduction of these therapies into clinical practice, considerations must be given to their integration with current standard-of-care chemotherapy. Additionally, comprehending the mechanisms of chemotherapy resistance in PDAC is imperative for managing this systemic ailment. Finally, as PDAC treatment strategies hopefully progress, attention should also be directed toward initiatives aimed at enhancing the quality of life for patients, such as addressing cancer-associated cachexia. We anticipate that the forthcoming decade will witness a proliferation of precision oncology approaches for this challenging cancer, benefiting an expanding cohort of patients.

## Figures and Tables

**Table 1:** Low frequency occurring mutations in PDAC patients (modified from Regel et al. [19]).

Functions	Incidence	Genes
Epigenetic Regulators	35%	ARID1A, KMT2C, KMT2D, KDM6A, SMARCA4, SETD2, ARID2, PBRM1, HDAC1, CREBBP, SETDB1, SETD1B, EP300, JARID2, KMT2A, SMARCA1, SMARCA2, KDM5C, SETBP1, KDM2B, ARID3C, DNMT3B, DNMT1, ARID4A, KDM5A, SMARCB1, SMARCD1, SETD1A, KAT6B
DNA Damage Response	9%	BRCA1, BRCA2, PALB2, ATM, ATR, MLH1, MSH2, MSH6, RPA1, STK11, FANCA, FANCC

**Table 4:** Targeted therapies in PDGF.

Drug	Target	References
Sunitinib	PDGFRβ)	[150,151]
Nintedanib	PDGFRα/β	[152,153]
Imatinib	PDGFR	[154]
Masitinib	PDGFR-3	[156]
TK1258 (Dovitinib)	PDGFB	[158]

**Table 5:** Summary of representative recent clinical trials (2019–2023) involving targeted therapies.

Clinical Trials Identifier	Targeted Therapy	Mechanism of Action
NCT03351296	Bevacizumab	Vascular endothelial growth factor (VEGF) inhibitor
NCT02259725	Regorafenib	Multi kinase inhibitor
NCT05934331	Toripalimab	Monoclonal antibody against programmed cell death protein 1 (PD-1)
NCT05580445		
NCT06111274		
NCT05862324	Activated T-lymphocyte cell therapy	Anti-tumoural immunity activation
NCT04137536		
NCT03269526		
NCT02311361	Tremelimumab	Monoclonal antibody against cytotoxic T-lymphocyte-associated protein 4 (CTLA-4)
NCT02305810	Everolimus	
NCT01088815	Hedgehog inhibitor	Inhibitor of the Hedgehog pathway
NCT02501902	Palbociclib	CDK4/6 inhibitor
NCT03558945	neoantigen peptide-based vaccines	Vaccines
NCT04161755	mRNA vaccines	
